# Postoperative morbidity and mortality of patients with COVID-19 undergoing cardiovascular surgery: an inverse propensity-weighted study: Postoperative outcomes of patients undergoing cardiovascular surgery during the COVID-19 pandemic

**DOI:** 10.1186/s12871-024-02445-5

**Published:** 2024-03-09

**Authors:** Fei Xu, Yunbo Bai, Fang Xie, Daqi Liu, Zhaoqi Wang, Sheng Wang

**Affiliations:** Department of Anesthesiology, Beijing Anzhen Hospital, Capital Medical University, Beijing, China

**Keywords:** COVID-19, Cardiovascular surgery, Morbidity, Mortality

## Abstract

**Background:**

To evaluate the postoperative morbidity and mortality of patients undergoing cardiovascular surgery during the 2022 nationwide Omicron variant infection wave in China.

**Methods:**

This retrospective cohort study included 403 patients who underwent cardiovascular surgery for the first time during the 2022 wave of the pandemic within 1 month. Among them, 328 patients were preoperatively diagnosed with COVID-19 Omicron variant infection during the pandemic, and 75 patients were negative. The association between Omicron variant exposure and postoperative prognosis was explored by comparing patients with and without COVID-19 exposure. The primary outcome was in-hospital death after cardiovascular surgery. The secondary outcomes were major postoperative morbidity, including myocardial infarction (MI), acute kidney injury (AKI), postoperative mechanical ventilation hours, ICU stay hours, and postoperative length of stay. The data were analyzed using inverse probability of treatment weighting (IPTW) to minimize bias.

**Results:**

We identified 403 patients who underwent cardiovascular surgery, 328 (81.39%) had Omicron variant infections. In total, 10 patients died in the hospital. Omicron variant infection was associated with a much greater risk of death during cardiovascular surgery after adjustment for IPTW (2.8% vs. 1.3%, adjusted OR 2.185, 95%CI = 1.193 to 10.251, *P* = 0.041). For major postoperative morbidity, there were no significant differences in terms of myocardial infarction between the two groups (adjusted OR = 0.861, 95%CI = 0.444 to 1.657, *P* = 0.653), acute kidney injury (adjusted OR = 1.157, 95%CI = 0.287 to 5.155, *P* = 0.820), postoperative mechanical ventilation hours (B -0.375, 95%CI=-8.438 to 7.808, *P* = 0.939), ICU stay hours (B 2.452, 95%CI=-13.269 to 8.419, *P* = 0.660) or postoperative stay (B -1.118, 95%CI=-2.237 to 1.154, *P* = 0.259) between the two groups.

**Conclusion:**

Perioperative COVID-19 infection was associated with an increased risk of in-hospital death among patients who underwent cardiovascular surgery during the Omicron variant wave of the pandemic.

## Introduction

Since the outbreak of COVID-19, more than 160 countries have the disease a pandemic, which has had an enormous impact on the global medical system and economic losses [1, 2]. On December 6th ,2022, the Notice on Further Optimizing the Implementation of COVID-19 Prevention and Control Measures (referred to as the “Ten New Guidelines”) [3] announced the beginning of the Omicron wave and that all levels of medical institutions should ensure normal medical services and medical convenience for patients in China. Compared with the Delta variant or the original strain, Omicron variant exhibited considerable transmissibility but decreased disease severity [4]. . Additionally, the majority of the population had received the COVID-19 vaccine. Accordingly, the focus on perioperative management of surgical patients with a history of Omicron infection changed from early nosocomial infection control and disease prevention to postoperative prognosis improvement.

Guidelines for the management of surgical patients were issued during the pandemic [5, 6]. However, the available information was not up-to-date and did not involve the Chinese population regarding the less virulent Omicron variant that caused a nationwide wave in China. Owing to a lack of evidence, the perioperative management of surgical patients with Omicron infection in China is currently challenging for surgeons and anesthesiologists.

Cardiovascular surgery patients are a particularly vulnerable population because perioperative concomitant infection is a burden associated with adverse outcomes [7]. The impact of COVID-19 infection on postoperative morbidity and mortality during cardiovascular surgery needs to be identified to enable surgeons and patients to make evidence-based decisions during pandemics.

The aim of this study was to assess the outcomes of cardiovascular surgery among patients who were positive for COVID-19and negative for COVID-19 during the first month of the Omicron wave in China. The information in this study has important implications for patients considering cardiovascular surgery during the COVID-19 pandemic.

## Materials and methods

### Patients and study design

This was a retrospective analysis of data collected from the largest volume of cardiac surgery hospital data in China. In total, 567 consecutive patients were examined from December 6, 2022, to January 6, 2023, for the first time during the Omicron wave.

The patients underwent one of five surgical procedures: coronary artery bypass graft (CABG), valvular surgery, CABG and valvular combined surgery, aortic dissection surgery or congenital surgery. All surgeries were open cardiothoracic surgeries. All patients were 18 + years of age. The exclusion criteria were reoperation, emergency surgery or minimally invasive surgery. A total of 403 patients met the inclusion criteria. Patients were classified as COVID-19 positive if they had a record of a positive COVID-19 test 14 days before surgery. We compared the postoperative outcomes between COVID-19 positive patients and COVID-19 negative patients during the pandemic. All the extracted patients were from Chinese ethnic population. Dedicated staff were responsible for the collection, completeness and submission of the hospitalization materials. This study was approved by the Ethics Committee of Beijing Anzhen Hospital (No. 2023164X).

### Study outcomes

The primary outcome of this study was in-hospital death after surgery which was determined based on hospital medical records.

The secondary outcomes included the incidence of major postoperative morbidity, including myocardial infarction (MI); and acute kidney injury (AKI); the duration of mechanical ventilation time, the length of intensive care unit (ICU) stay; and the length of postoperative stay.

Myocardial infarction was defined as an absolute increase in cardiac troponin levels (from baseline) ≥ 75 times the upper reference limit plus 1 of the following criteria: ①ischemic ST-T changes; ②New significant Q waves ③Imaging evidence showed loss of myocardium or abnormal local wall motion [8]. . Acute kidney injury was defined by the Kidney Disease: Improving Global Outcomes (KDIGO) criterria [9] as plasma creatinine levels ≥ 1.5 times greater than baseline plasma creatinine levels or a 26.5umol/L increase in plasma creatinine levels within 48 h after surgery compared with baseline levels.

### Statistical analysis

Descriptive variables are presented as the means ± standard deviations; the t test was used to assess differences. Categorical variables are shown as frequencies (percentages), and comparisons were made with chi-square tests. Two-tailed *P* values ≤ 0.05 were considered to indicate statistical significance. All the statistical analyses were performed using SAS 4.2.0 (SAS Institute Inc., Cary, NC, USA).

To adjust for the impact of treatment selection bias and potential confounders in this study, propensity scores were calculated via multiple logistic-regression analysis, and inverse probability of treatment weighting was employed. We applied probability weights equal to the inverse propensity score for positive patients, and probability weights were calculated as the inverse of 1 minus the propensity score for negative patients. A separate propensity score for positive versus negative patients was derived for each comparison. The odds ratios (ORs) and estimated coefficients (B) for postoperative outcomes were calculated, and 95% confidence intervals were used to show the effect sizes of comparisons.

## Results

A total of 403 patients were ultimately included in this original study. Among them, 328 were positive for COVID-19 during the preoperative period, and 75 patients were negative. The two groups had similar baseline characteristics with respect to age, sex, BMI, hypertension, diabetes mellitus, myocardial infarction, chronic heart failure, creatinine, ejection fraction and NYHA class. However, more people in the positive group had higher hyperlipidemia (35.1% vs. 17.3%, *P* = 0.005) and diabetes mellitus (29.3% vs. 17.3%, *P* = 0.036) (Table 1).

**Table 1:** Demographic and clinical characteristics of the participants

Characteristics	Entire cohort		*p*
	COVID-19 Positive (*n* = 328)	COVID-19 Negative (*n* = 75)	
Age	57.57 ± 11.42	57.82 ± 11.10	0.501
Female,n(%)	89 (27.1)	24 (32.0)	0.482
BMI	25.57 ± 7.75	24.92 ± 3.48	0.479
Hypertension	173 (52.7)	30 (40.0)	0.062
Diabetes mellitus	96 (29.3)	13 (17.3)	0.036
Hyperlipidemia	115 (35.1)	13 (17.3)	0.005
COPD	5 (1.5)	0	0.619
Peripheral vascular disease	15 (4.6)	3 (4.0)	NA
Cerebrovascular events	47 (14.3)	11 (14.7)	NA
Myocardial infarction	45 (13.7)	12 (16.0)	0.743
Chronic heart failure	21 (6.4)	1 (1.3)	0.144
Creatinine	84.94 ± 70.18	85.28 ± 67.47	0.383
Ejection fraction	58.20 (8.64)	56.97 (9.30)	0.277
NYHA class			0.936
I	111 (33.8)	27 (36.0)	
II	206 (62.8)	45 (60.0)	
III	11 (3.3)	3 (4.0)	
Surgery type			0.102
CABG	191(58.2)	51(68.0)	
CABG + valvular surgery	17(5.2)	0(0)	
Valvular surgery	66(20.1)	9(12.0)	
Aortic dissection repair	29(8.8)	7(9.3)	
Other	25(7.6)	8(10.7)	

BMI = body mass index; COPD = chronic obstructive pulmonary disease; Values are n (%) for categorical variables

### Primary outcomes

In total, the overall in-hospital mortality rate was 2.48% (10/403). In the negative group, only one patient who underwent CABG died from postoperative acute myocardial infarction. In the positive group, 9 patients died- 3 patients who underwent CABG ,2 patients who underwent CABG and valvular surgery, 2 patients who underwent valvular surgery only and 2 patients who underwent aortic dissection surgery. In-hospital deaths associated with Omicron infections were caused mainly by direct life-threatening respiratory failure and toxic shock.

### Secondary outcomes

The major postoperative morbidities during the Omicron wave were compared between COVID-19 positive patients who underwent cardiovascular surgery and negative patients using inverse probability weighting. Tables 2 and 3 show the contingency tables for each major complication with *p* values.

**Table 2:** Postoperative outcomes according to COVID-19 status

Variable	Entire cohort		Adjusted by inverse probability of treatment weights		
	COVID-19 Positive	COVID-19 Negative	Adjusted OR	95% CI	*p* Value
	*N* = 328	*N* = 75			
In-hospital death	9(2.8)	1(1.3)	2.185	1.193–10.251	0.045
Myocardial infarction	19(5.8)	3(4.0)	0.861	0.444–1.657	0.653
Acute kidney injury	8(2.4)	1(1.3)	1.175	0.287–5.155	0.820

OR, odds ratio; CI, confidence interval

**Table 3:** Postoperative outcomes according to COVID-19 status

Variable	Entire cohort		Adjusted by inverse probability of treatment weights		
	COVID-19 Positive	COVID-19 Negative	B	95% CI	*p* Value
	*N* = 328	*N* = 75			
Postoperative mechanical ventilation hours	27.10 ± 45.44	25.39 ± 54.86	-0.375	-8.438-7.808	0.939
ICU stay hours	41.40 ± 65.22	39.01 ± 60.12	2.452	-13.269-8.419	0.660
Postoperative hospital length of stay (days)	10.78 ± 4.43	10.71 ± 8.08	-1.118	-2.237-1.154	0.259

There was no significant difference in terms of myocardial infarction (adjusted OR = 0.861, 95%CI = 0.444 to 1.657, *P* = 0.653); acute kidney injury (adjusted OR = 1.157, 95%CI = 0.287 to 5.155, *P* = 0.820); postoperative mechanical ventilation hours (B -0.375, 95%CI=-8.438 to 7.808, *P* = 0.939); ICU stay hours (B 2.452, 95%CI=-13.269 to 8.419, *P* = 0.660) or postoperative length of stay (B -1.118, 95%CI=-2.237 to 1.154, *P* = 0.259) between the two groups.

Even though there was no significant difference in postoperative morbidity between the two groups, the tendency was greater in the positive group.

## Discussion

This is the first and largest study to estimate the effect of the Omicron wave on patients undergoing cardiovascular surgery in China. We sought to confirm that the outcomes of COVID-19 positive patients were comparable to those negative patients and to determine the best result for cardiovascular surgery during the pandemic. In this study, the incidence rates of postoperative morbidity and mortality were high among patients with a history of Omicron infection.

Preoperative COVID-19 was previously shown to be associated with significantly increased risks of morbidity and mortality, and consensus recommendations were made to delay elective surgery for 7 weeks after SARS-CoV-2 infection [10, 11]. However, such evidence represents the early wave of the COVID-19 pandemic, which was triggered by the ancestral variant, as opposed to the less virulent Omicron variant that caused the 2022 nationwide wave of infections in China [12]. Consequently, earlier evidence may not be applicable for guiding current practices during this pandemic wave in China [13].

China deployed successful multilayer nonpharmaceutical interventions (NPIs), and the majority of the population experienced an Omicron infection for the first time during the 2022 wave of the pandemic within 1 month [14]. The likely cause of this was the absence of previous epidemics, with the majority of infected patients experiencing the first course of the disease. This necessitated a prompt response in terms of perioperative management, particularly in the Chinese population. Therefore, we explored the feasibility of this mitigation strategy during the Omicron wave for perioperative patient management to facilitate decision-making while minimizing the disease burden.

At one month after the onset of the Omicron wave, a cohort of 403 individuals who underwent cardiovascular surgery procedures was collected, which represented a substantially decreased number of cardiac surgeries during the pandemic, with a 54% decrease in surgical volume compared with the previous year at our hospital. This means that most elective surgical interventions were deferred during the pandemic. Our research showed that the outcomes of cardiovascular surgery among patients who had COVID-19 during the perioperative period were significantly poor outcomes of increased mortality during hospitalization. The mortality rates for the positive and negative patients were 2.8% and 1.3%, respectively, which were statistically significant. For postoperative major morbidity, the positive group had a high rate, even though the difference was not statistically significant.

CABG surgeries and vascular surgeries were still the most common procedures performed during the pandemic [15]. In our research, the in-hospital mortality rate of CABG in the positive group was 1.57% (3/191), which was higher than that of 1.32% in the pre-COVID-19 era in our hospital [16]. Life-threatening disease during surgical repair of aortic dissection in patients with confirmed COVID-19 may result in fatal outcomes. In-hospital mortality was 8.16% (4/49) higher than that in the pre-COVID-19 era(6.1%) in our hospital [17]. Aortic dissection repair is an enormous challenge given the technical difficulty and complexity of this procedure as well as the cardiopulmonary damage caused by COVID‐19 during the pandemic.

The Omicron variant had lower pathogenicity, resulting in considerable lower rates of severe illness and death due to less severe disease and an improved clinical profile compared to those of the original strain. Additionally, the majority of the population was completely vaccinated. On the other hand, Omicron’s increased transmissibility is due to its rapid replication in the upper airways and increased attachment of the surface spike protein to angiotensin-converting enzyme 2 (ACE-2) receptors [18, 19]. There is abundant evidence suggesting that ACE-2 receptor-mediated endothelial dysfunction can cause severe myocardial injury [20–22]. Therefore, despite the decreased disease severity experienced with the Omicron variant, it continues to pose a major threat to cardiometabolic demand with resultant pressure on the health care system, as illustrated by the patients’ high incidence of in-hospital death. An updated guideline from the United Kingdom recommended that patients avoid elective surgery within 2 weeks after SARS-CoV-2 infection [23].

This study explored the influence of the Omicron variant on the postoperative prognosis of patients who underwent cardiovascular surgery. The risk of in-hospital death was higher among COVID-19-positive patients.

### Conclusion

Aa many regions worldwide continue to experience the spread of COVID-19, it is necessary to estimate the risk associated with cardiac surgery during a pandemic. Preoperative COVID-19 positivity during thepandemic was associated with an increased risk of in-hospital death among patients undergoing cardiac surgery. However, for patients with severe multivessel coronary or aortic dissection requiring emergency procedures, even though there is an increased likelihood of mortality compared with the pre-COVID-19 era, delay would more likely result in patient harm.

## Data Availability

The datasets used and/or analyzed during the current study are available from the corresponding author upon reasonable request.
